# Nationwide Awareness Campaign and Call for Dental Screening for Hereditary Hemorrhagic Telangiectasia in Germany

**DOI:** 10.1155/2023/8737727

**Published:** 2023-02-11

**Authors:** Urban W. Geisthoff, Frank Hölzle, Boris A. Stuck, Joachim Jackowski, Catherine Hand Goetz, Christina Grabowski, Freya Droege

**Affiliations:** ^1^VASCERN HHT Reference Centre, Department of Otorhinolaryngology, Head and Neck Surgery, University Hospital Marburg, Philipps-Universität Marburg, Baldingerstrasse, Marburg 35043, Germany; ^2^Morbus Osler-Selbsthilfe e.V. (German HHT Self-Help Group), Berlin, Germany; ^3^Department of Oral and Maxillofacial Plastic Surgery, University-Hospital Aachen, RWTH Aachen, Pauwelsstraße 30, Aachen 52074, Germany; ^4^Department of Oral Surgery and Dental Emergency Care, School of Dentistry, Faculty of Health, Witten/Herdecke University, Alfred-Herrhausen-Strasse 44, Witten 58455, Germany; ^5^Arkansas College of Osteopathic Medicine, Fort Smith, AR, USA; ^6^VASCERN HHT Reference Centre, Department of Otorhinolaryngology, Head and Neck Surgery, Essen University Hospital, University Duisburg-Essen, Hufelandstrasse 55, Essen 45122, Germany

## Abstract

**Objectives:**

Hereditary hemorrhagic telangiectasia (HHT) is a rare disorder encompassing facial and oral telangiectasias and visceral vascular malformations (VMs). Pulmonary VMs can lead to paradoxical embolism of thrombi or bacteria, e.g., due to dental procedures. Early detection can reduce morbidity and mortality and is recommended. However, diagnosis is often delayed for decades. Our study is assessing the feasibility and effect of a nationwide awareness campaign for early diagnosis of HHT addressing all dentists in Germany.

**Methods:**

In 2018, one article and two reminders about HHT were published in a nationwide awareness campaign. As a proxy for the effectiveness of the campaign, researchers measured the number of first-time inquiries from patients and physicians about HHT documented by the German HHT self-help group from September 2016 until September 2019.

**Results:**

A total of 411 first contacts with the German self-help group were documented, mainly via Internet platforms (Internet forum (*n* = 130) and Facebook® (*n* = 189)). For 9% of those patients (*n* = 36/411), the physician or dentist (physician: (*n* = 31/36, 86%; dentist: *n* = 5/36, 14%) informed patients about the disease HHT and the self-help group. Before publishing the first article about HHT, no dentist referred patients to the German self-help group; afterwards, 5 patients received information about HHT from their dentist and contacted the patient organization for the first time. After each publication in June, September, and December 2018, the number of new contacts increased. Contacts via phone and e-mail had the highest relative increase.

**Conclusions:**

The repeated call for dental screening for HHT in Germany led to increased awareness of this rare disease; more patients with possible HHT received information about the condition. The authors conclude that targeted campaigns may contribute to a shorter diagnostic latency resulting in increased quality of life and life expectancy in HHT. This trial is registered with CT03549949.

## 1. Introduction

**Figure 1:**
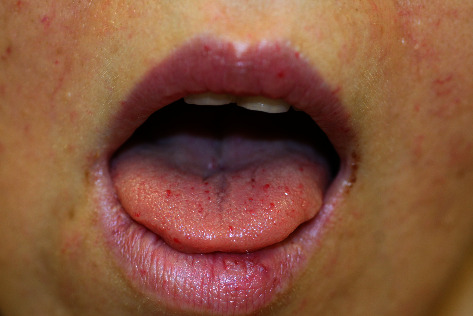
Typical telangiectasia of the skin and mucosa in a patient with HHT. HHT = hereditary hemorrhagic telangiectasia.

Hereditary hemorrhagic telangiectasia (HHT) results in vascular malformations (especially capillary and arteriovenous), involving multiple tissues and organs. It is inherited as autosomal dominant trait [1]. With an estimated prevalence of 1-2 individuals per 10000, the disease is a “relatively frequent” rare disorder [2, 3]. Patients suffer from recurrent bleedings and problems arising from blood shunting due to visceral vascular malformations. Most patients develop recurrent epistaxis and multiple typical telangiectasias of the skin or mucosa, in particular, on their face, oral mucosa, and lips (Figure 1). We know from patient testimonies and a few case reports that gingival telangiectasias can bleed as a result of manipulations, e.g., brushing or flossing of the teeth or probing during dentist visits [4, 5]. Visceral vascular manifestations most commonly affect patients' lungs, livers, and brains [6]. Pulmonary arteriovenous malformations (PAVMs) can lead to disturbed filter function of the lungs resulting in abscesses and infarctions of visceral organs like the brain, liver, and spleen [7–10]. Therefore, in patients with likely HHT, screening for PAVMs is recommended [11, 12]. The diagnosis of HHT is based on genetic testing and/or the International Clinical Diagnostic Criteria, also known as the Curaçao Criteria. Genetic testing can reveal the diagnosis HHT in most cases, but not all disease-causing mutations have been detected [13]. Using the Curaçao Criteria based on patients' family history and the history of epistaxis, telangiectasia, and visceral lesions, the diagnosis HHT can be established. The presence of at least three out of these four clinical criteria is required in order to diagnose HHT [6, 11].

Dentists, as health care professionals, routinely examine the mucosa of the mouth. The detection of typical telangiectasias on the patients' oral mucosa and perioral skin during these examinations is likely. By asking additional questions about the family history and recurrent epistaxis, the diagnosis of HHT could be considered. In this study, the results of a nationwide German HHT awareness campaign and call for dental screening were analysed.

Previous studies have shown that both early HHT diagnosis and screening for pulmonary involvement, at least, reduced morbidity and mortality in patients with HHT. Referral to an interdisciplinary centre that is specialized in HHT is advantageous for these tasks [14, 15]. Patients with HHT and pulmonary arteriovenous malformations (and there where this has not been screened yet) should be informed about the recommendation to use an antibiotic prophylaxis for certain medical procedures [16–18]. This important information was published in articles and reminders for dentists. Furthermore, this information was printed on special patient cards and distributed by the German HHT patient organization. Adoption of these measures by patients with HHT can help prevent complications such as strokes and abscesses [11], which was a major goal of our awareness campaign.

## 2. Methods

From September 2016 until September 2019, the German HHT self-help group documented all first inquiries from patients and physicians about HHT. The self-help group was contacted directly via email or phone. Additionally, a board member of the German HHT self-help group provided data from an Internet forum (https://www.morbusosler.info/) and a closed Facebook® group on HHT (https://www.facebook.com/groups/1889282361305763/).

In June 2018, a case report of a patient with HHT and PAVM developing a liver abscess after professional tooth cleaning together with an explanation and instruction on disease management in HHT was published in the journal of the German dental association and the Federal Association of Fund Dentists of Germany [16]. This journal is distributed to all dentists in Germany and is a set book for them with an average of 77000 readers [19]. Three and six months later, a reminder was published in the same journal [17, 18]. In these three articles, dentists were asked to pay attention to typical telangiectasias of the oral mucosa and facial skin and–upon discovery–to ask the patients if they or other relatives have nosebleeds. If HHT is suspected, it was advised to recommend that the patient should get in contact with a medical centre to clarify a potential diagnosis. The self-help group was recommended as a way for patients to find the appropriate medical centre. The phone number and email address of the self-help group were given for this purpose. Technical problems led to a loss of documentation on the number of emails the German self-help group received from September 2016 to April 2018, so that only the email-documentation from April 2018 until September 2019 was available for analysis. However, the documentation for phone calls was complete.

Data on type and time of contacts were collected by the German self-help group and sent to our research group in an anonymized form. Statistical analyses were performed with Microsoft Excel (Microsoft Cooperation, version 16.0).

## 3. Results

**Figure 2:**
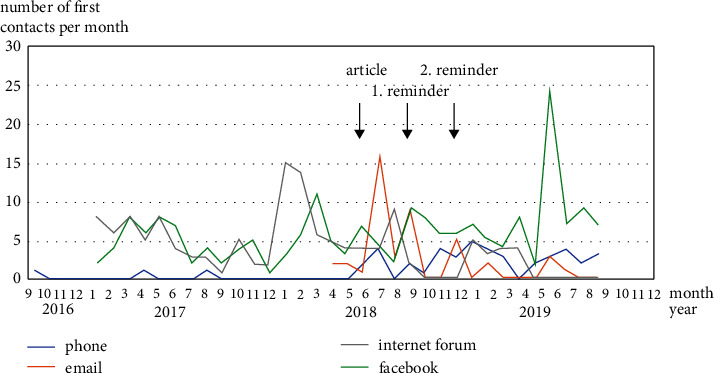
First contacts to the German self-help group per month. Distribution of all 411 first contacts to the German self-help group from 09/2016 to 09/2019 by different communication channels and in relation to the publications of the awareness campaign. After publication of the article the number of first contacts to the German self-help group via phone calls and emails increased. Mentioning the publications of the awareness campaign in the independent (patient led) closed facebook® group also led to a higher number of activities. Due to mainly technical reasons the documentation for the whole period is only complete for first contacts by phone. The period of documentation is limited from 04/2018–09/2019 due to a hard drive problem of the self-help group in spring 2018. Documentation of the new contacts by internet forum and facebook® started in 1/2017, however, it was stopped preterm for the internet forum due to a crash of the forum in spring 2018.

Between September 2016 and September 2019, a total of 411 first contacts were documented by the German HHT self-help group via Internet platforms (Internet forum (*n* = 130), Facebook® (*n* = 189)), phone (*n* = 45), and email (*n* = 43; Figure 2).

In 91 cases (91/411, 22%), the source of information about the self-help group was known. Data on referrals by physicians were only available for 36 of 91 (40%) contacts during the study time. Most patients received information about HHT and the German self-help group via their general practitioner (16/36, 44%) and otorhinolaryngologist (9/36, 25%). In 5 patients, their dentists recognized the diagnosis HHT (5/36, 14%); all these 5 contacts occurred after the publication of the first article. In 8% (3/36), their radiologists, in 6% (2/36), their pulmonologists, and in 3% (1/36), their haematologist informed the patients (see supplementary data Figure S1); 48% of the patients (*n* = 44/91) did independent Internet research about HHT, 9 patients (9/91, 10%) were informed by family members, 2 patients (2/91, 5%) were informed by friends and members of another self-help group (pulmonary hypertension), and 1 patient read an article about HHT (1/91, 1%) [20].

**Figure 3:**
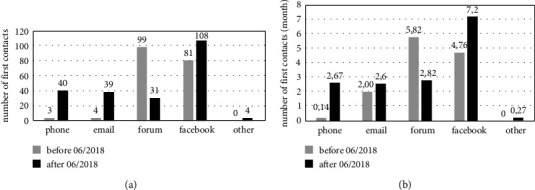
Number of first contacts via different communication channels for different calculations and time periods. This graph shows the number of all first contacts documented between September 2016 and 2019 (a) and the average number of first contacts per months of documentation (b). There was a relevant increase in phone contacts after publishing the article. Documentation periods (for explanation see also method section): phone: 09/2016–05/2018 and 07/2018–09/2019; email: 04/2018–05/2018 and 07/2018–09/2019; forum: 01/2017–05/2018 and 07/2018–04/2019; facebook: 01/2017–05/2018 and 07/2018–09/2019. In total 4 patients got into contact with the German self-help group via other communication channels (“other”: 2x via the facebook^®^ homepage of the self-help group for patients with pulmonary hypertension, 1x indirect via friends who were contacting the German self-help group via facebook^®^ and 1x directly via the general practitioner).

A total of 187 contacts to the self-help group were made between September 2016 and May 2018 (187/411, 45%) before the first dental awareness campaign publication. Seven of those contacts were via phone or e-mail (7/187, 4%). After each publication in June, September, and December 2018, the number of contacts increased. Contacts via phone and email (these communication options were recommended in the publications) had the highest relative increase (number of contacts after 06/2018 divided by number of contacts between 9/2016 and 9/2019); all communication channels: 222/411, 54%; phone: 40/43, 93%; email: 39/43, 91%; Internet forum: 31/130, 24%; Facebook®: 108/189, 57% (Figures 3 and S2 in the supplementary data).

## 4. Discussion

To our knowledge, this study describes the first awareness campaign for HHT based on the inclusion of dentists. Every year, more than 70% of the German population receive a professional tooth cleaning and examination by their dentists as it is supported by the German public health insurances [21, 22]. Therefore, our approach had the potential for a nationwide population-based campaign. In HHT, over 80% suffer from visible symptoms, like muco-/cutaneous telangiectasia, [6, 23–25] which could be detected by their dentists, who are primary health care professionals treating patients of all ages and sexes. Nosebleeds, the presence of telangiectasias, and/or a positive family history are sufficient to raise suspicion for HHT in the setting of a dental practice, resulting in the appropriate referral for further evaluation. Early diagnosis and treatment achieved–like in this case through dental screenings—can lead to improved quality of life for HHT patients [15, 26].

The results of the awareness campaign demonstrated a positive effect, that is, our nationwide dental awareness campaign was associated with a higher frequency of persons contacting the self-help group for the first time. However, the 10% increase in contacts for an equivalent time interval seems to be relatively small (187 first contacts for the period between September 2016 to May 2018 in comparison to 222 contacts for the period between July 2018 to September 2019). Various explanations exist for the limited impact of the campaign. Our approach targeted dentists. In Germany, dentists normally do not write referrals which may have contributed to the limited effect. Only 9% of patients indicated that they were informed by their dentists or other physicians on the possibility of HHT. Before the campaign, no dentist referred patients to the German self-help group; afterwards, at least 5 patients received information about HHT by their dentists and mentioned this when they contacted the patient organization for the first time. It is probable that this effect was related to the awareness campaign, which we interpreted as a limited success. However, it is interesting that 44% of patients stated that they were informed mainly by their general practitioners and only 14% by dentists. A higher rate of referrals by dentists was expected. The authors were not able to identify the reason for this but one possibility is that dentists first referred to the general practitioner who then recommended that patients contact the self-help group.

Awareness campaigns for rare diseases like HHT are necessary in order to reduce the diagnostic latency and improve the medical care of these patients [27, 28]. In general, many problems in treating patients with rare diseases result from the lack of awareness or experience among healthcare professionals, low motivation, and barriers to changing practice or policies. One approach to increase awareness of and familiarity with rare diseases in healthcare is to target the postgraduate education of gatekeeper care providers, like dentists or primary care physicians [29, 30]. Health promotion activities that focus on large populations such as smoking cessation campaigns may affect the health of many individuals which encourages engagement by primary care physicians and government agencies. However, mainly due to the lack of exposure or awareness of primary care providers or politicians, the incentive to change practice or policy might be limited for rare diseases [31]. The “Shit Happens” campaign is an example of a parents-oriented research project for patients with the rare condition called the Hirschsprung's disease. The campaign demonstrated that social media can be a powerful and responsive tool to connect families with rare diseases [32].

To tailor the HHT awareness campaign to dentists as the target audience, we studied approaches taken by earlier campaigns targeting dentists. These campaigns were mainly based on online tools and resulted in low response rates amongst dentists [33, 34]. Therefore, we developed a different approach by publishing repeated articles in a set book for all dentists in Germany. In our awareness campaign, a case report about HHT together with instructions about disease management of HHT was published online and in print in the journal of the German dental association and the Federal Association of Fund Dentists of Germany which is received by every dentist in Germany. This journal is a set book for all dentists in Germany with a total circulation of 77621 copies in 2017. After publication of the article, the number of first contacts to the German self-help group via phone calls and emails increased, especially when comparing the time periods two months before and after the first publication. Only these two means of communication were suggested in the publication, and subsequently, the increase in these routes was higher than in other channels like Facebook® or Internet inquiries, suggesting that the rise in new contacts might be associated with our campaign. Interestingly, almost half of the patients answering to this question did some Internet research about HHT and the German self-help group to access information on the disease. This shows that it is very difficult to specifically ascribe those changes only to the dentist awareness campaign, without considering the contribution of the increased knowledge and awareness by patients and clinicians (also from different specialties) that may occur during the campaign period. It also underscores the fact that the Internet has become a very popular source of health-related information for patients [35]. Likewise, many healthcare professionals use web-based tools for obtaining health-related information; however, defective synthesis of the available information can lead to diagnostic error [36]. A potential aim for future studies might be to identify the number of dentist-specific referrals, due to awareness campaigns, more accurately.

Data were provided by the German HHT self-help group. Due to technical problems, the documentation of the different communication channels for the whole period is only complete for first-time contacts by phone. It should be noted that another limitation of the study was that neither patients' details (i.e., sex, age, and clinical symptoms) nor a follow-up of completed screening or necessary therapies could be documented due to data protection regulations. However, the cooperation of the self-help group for HHT and medical professionals during this study demonstrates the important role that patient involvement plays in the healthcare system [37, 38].

The article on HHT was published in June 2018. On the beginning of July 2018, the new website of the German HHT self-help group was launched. Therefore, the effect of the new homepage might have been a confounding factor. However, a new website several years ago did not increase the number of phone contacts at that time.

The development of a table showing oral manifestations and basic information, not just for HHT but also for other rare diseases, will be a task for our future work in order to facilitate the diagnostic process and to inform dentists about treatment options of different rare diseases.

## 5. Conclusion

This study demonstrates that a nationwide awareness campaign and call for dental screening for HHT in Germany lead to an increased number of persons with possible HHT being informed about the disease. This might contribute to early diagnosis and treatment in HHT and consequent improvements in morbidity and mortality among HHT patients.

## Data Availability

The datasets are available from the corresponding author on reasonable request.

## Abbreviations

HHT: Hereditary hemorrhagic telangiectasia
PAVM: Pulmonary arteriovenous malformations
*N*: Number of patients/contacts
VM: Vascular malformations.

## Supplementary Materials

Supplementary Materials Figure S1: Different specialists referring patients with HHT to the German self-help group. Data on referrals by physicians were only available for 36 of 411 contacts during the study time. Most patients received information about hereditary hemorrhagic telangiectasia (HHT) and the German self-help group via their general practitioner (number of patients (*N*) = 16/36, 44%) and otorhinolaryngologist (*N* = 9/36, 25%). In 5 patients, their dentists recognized the diagnosis HHT (*N* = 5/36, 14%), all these 5 contacts occurred after the publication of the first article. Figure S2:Number of first contacts via different communication channels directly before and after publishing the initial article. This graph demonstrates the number of first contacts two months directly before and after publishing the initial article in June 2018.
