# Supplementary material for: Nationwide Awareness Campaign and Call for Dental Screening for Hereditary Hemorrhagic Telangiectasia in Germany
Source: Int J Dent. 2023 Feb 11;2023:8737727. doi: 10.1155/2023/8737727 (PMC9938772; doi:10.1155/2023/8737727)
Supplement: Supplementary Materials — Figure S1: Different specialists referring patients with HHT to the German self-help group. Data on referrals by physicians were only available for 36 of 411 contacts during the study time. Most patients received information about hereditary hemorrhagic telangiectasia (HHT) and the German self-help group via their general practitioner (number of patients (N) = 16/36, 44%) and otorhinolaryngologist (N = 9/36, 25%). In 5 patients, their dentists recognized the diagnosis HHT (N = 5/36, 14%), all these 5 contacts occurred after the publication of the first article. Figure S2:Number of first contacts via different communication channels directly before and after publishing the initial article. This graph demonstrates the number of first contacts two months directly before and after publishing the initial article in June 2018. [file 8737727.f1.pdf]

Supplementary data:

Figure S1: Different specialists referring patients with HHT to the German self-help group

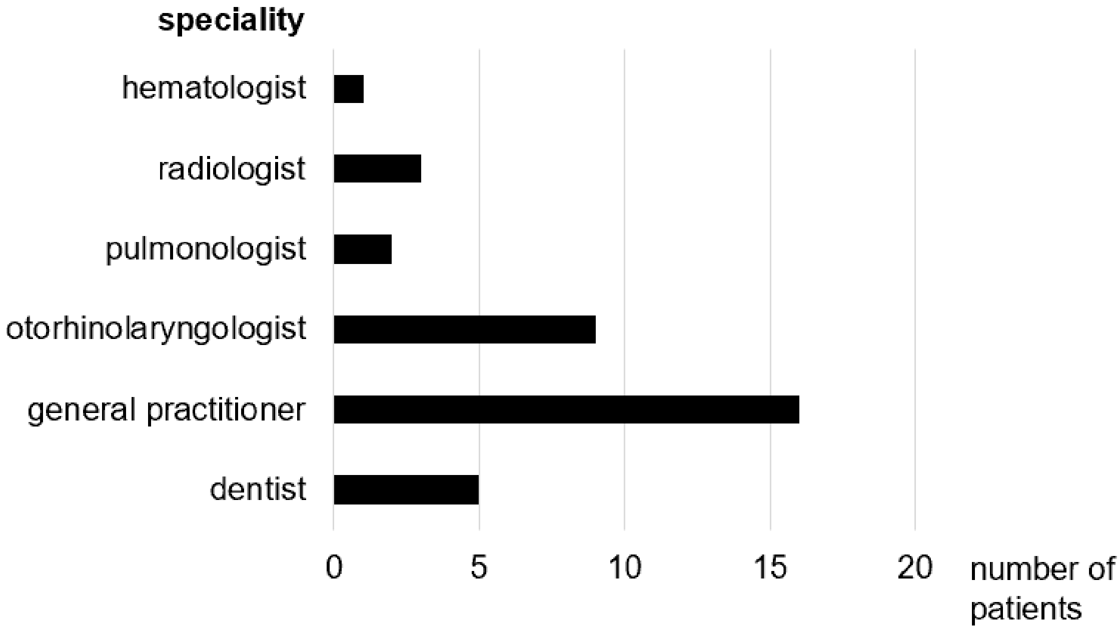

Data on referrals by physicians was only available for 36 of 411 contacts during the study time. Most patients received information about hereditary hemorrhagic telangiectasia (HHT) and the German self-help group via their general practitioner (number of patients (N) = 16/36, 44%) and otorhinolaryngologist (N = 9/36, 25%). In 5 patients their dentists recognized the diagnosis HHT (N = 5/36, 14%), all these 5 contacts occurred after the publication of the first article.

Figure S2: Number of first contacts via different communication channels directly before and after publishing the initial article

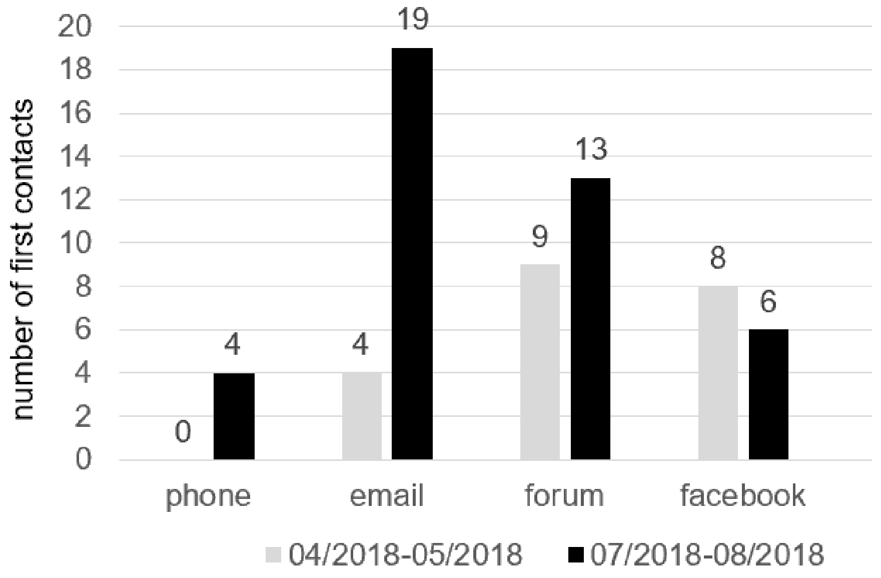

This Graph demonstrates the number of first contacts two months directly before and after publishing the initial article in June 2018.
